# Radiogenomic Analysis of Oncological Data: A Technical Survey

**DOI:** 10.3390/ijms18040805

**Published:** 2017-04-12

**Authors:** Mariarosaria Incoronato, Marco Aiello, Teresa Infante, Carlo Cavaliere, Anna Maria Grimaldi, Peppino Mirabelli, Serena Monti, Marco Salvatore

**Affiliations:** IRCCS SDN, Via E. Gianturco, 113, 80143 Naples, Italy; maiello@sdn-napoli.it (M.A.); tinfante@sdn-napoli.it (T.I.); ccavaliere@sdn-napoli.it (C.C.); agrimaldi@sdn-napoli.it (A.M.G.); pmirabelli@sdn-napoli.it (P.M.); smonti@sdn-napoli.it (S.M.); direzionescientifica@sdn-napoli.it (M.S.)

**Keywords:** radiogenomics, cancer, MR, texture analysis, microarray, NGS technologies, correlation matrix, molecular imaging, data mining

## Abstract

In the last few years, biomedical research has been boosted by the technological development of analytical instrumentation generating a large volume of data. Such information has increased in complexity from basic (i.e., blood samples) to extensive sets encompassing many aspects of a subject phenotype, and now rapidly extending into genetic and, more recently, radiomic information. Radiogenomics integrates both aspects, investigating the relationship between imaging features and gene expression. From a methodological point of view, radiogenomics takes advantage of non-conventional data analysis techniques that reveal meaningful information for decision-support in cancer diagnosis and treatment. This survey is aimed to review the state-of-the-art techniques employed in radiomics and genomics with special focus on analysis methods based on molecular and multimodal probes. The impact of single and combined techniques will be discussed in light of their suitability in correlation and predictive studies of specific oncologic diseases.

## 1. Introduction

Cancer diagnosis and classification are traditionally based on the histological examination of bioptic specimens. However, this approach presents challenges related to the invasive gathering of tissue, failures to distinguish between clinically relevant subtypes of cancer, and inter- and intra- observer variability [1]. In response to these drawbacks, new high-throughput platforms have emerged with the aim of better characterizing cancer at the molecular level, allowing an earlier diagnosis, better stratification, and more accurate prognosis than the histopathological approaches for targeted treatments. In this scenario, technological improvements in the field of imaging and molecular biology have led to “radiogenomics” or “imaging genomics” [2]. Literally, radiogenomics refers to the analytical processes aimed at correlating cancer imaging features (radiomics) with genomic data (genomics) [3].

**Figure 1:**
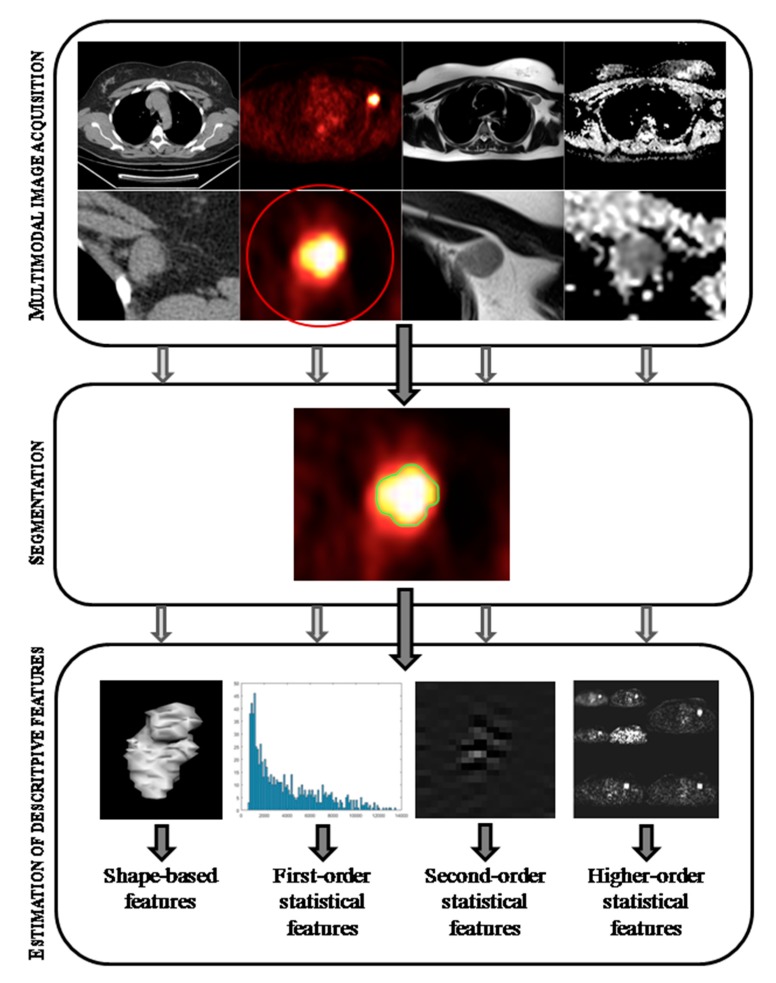
Schematic radiomic workflow. The process starts with the acquisition of common diagnostic images (Computed Tomography (CT), Positron Emission Tomography (PET), Magnetic Resonance (MR) see Section 2.1.1) and the identification of the lesions under investigation. The target regions are segmented (for the sake of simplicity, the process is shown for a single regions of interest (ROI) in the PET image only, as highlighted by the red circle) with the chosen approach (see Section 2.1.2). Finally, for each segmented region up to some hundreds of features, which are typically divided in shape-based, first-, second- and higher-order statistical features, can be computed (see Section 2.1.3).

The concept behind radiogenomics is the possibility of investigating the relationship between imaging, genomics, and clinical knowledge simply by looking at data, regardless of any qualitative interpretation; roughly speaking, by letting the data speak for themselves [4,5]. Therefore, radiogenomic approaches are extensively based on numerical calculus and computer science methods, allowing the management and analysis of a very large number of variables for each sample and modality. For instance, the image of an oncological lesion (as shown in Figure 1) is commonly rated by a radiologist using functional/morphological attributes (spiculated, enhanced, hyper-intense, focal, glucose avid), but the imaging study, itself, is a multivariate source of data; therefore, the main aim of radiomics is to extract meaningful information directly from data. How much radiogenomics has benefited from data processing techniques is clear, as it requires sophisticated algorithms and high-performance computing for the analytical processes. This latter consideration reveals the inherent multi-disciplinary nature of this emerging field, where radiologists, oncologists, biologists, and computer and statistical scientists work together to gain useful insights for personalized medicine. The wide development of this novel analytical approach is not limited to oncology; it is also successfully employed in neurology, where textural features related to oncological lesions can be replaced by morpho-functional features or connectivity of brain structures [6,7].

Although the basic concepts underlying the radiogenomics are essentially intuitive, the definition of their scope is rather controversial [8,9]. Two main reasons of ambiguity can be identified. The first one arises from the prefix radio that may be interpreted as referring to radiation leading to the radiogenomics meant as radiation genomics. This should aim to develop an assay able to predict which cancer patients might develop toxicity as a result of radiotherapy treatment and to identify genes possessing single nucleotide polymorphisms (SNPs) as possible biomarkers of radiation-induced adverse effect. The second reason lies in the suffix omics: it implies the generation of complex high-dimensional mineable data from each biological and imaging sample [10]. This requirement is not completely fulfilled by those radiogenomic studies that simply conduct correlations between low-dimensional data of imaging and genomic data.

Here, we report the most recent advances in molecular profiling research and technology applied to cancer, and their integration with imaging features. In the following sections, radiomic and genomic techniques will be treated separately, and will be followed by a subsection regarding their integration and data analysis approaches. In the Discussion section, the radiogenomics approach will be outlined for specific neoplastic diseases, separately analyzed by specifying the applied radiomic and genomic methods, also considering the goal of each study. Finally, in the last section, critical issues and future perspectives will be put forward.

In conclusion, this review aims to critically illustrate the most relevant radiomic and genomic techniques applied in the radiogenomics field, and how this new methodological approach was used in predictive and correlation oncological studies.

## 2. Methodologies

### 2.1. Radiomics

Radiomics refers to the comprehensive quantification of tumour phenotypes by the extraction of a large number of quantitative features from medical images. This high-throughput extraction of quantitative imaging features is the result of a workflow that is composed of three main steps (Figure 1):

- Acquiring the images
- Segmenting the regions of interest (ROIs)
- Estimating descriptive features.

Each point involves well-established medical image acquisition and processing techniques, but the integration of these procedures into a reliable and reproducible pipeline for radiogenomic analyses deals with complex and challenging issues in each step. In what follows, these steps will be separately described and discussed.

#### 2.1.1. Image Acquisition

A great advantage of radiomic analyses is their feasibility relative to conventional clinical scanners and imaging techniques. Indeed, the first step of the radiomic pipeline involves the acquisition of images that are typically part of diagnostic or treatment planning protocols for oncological patients [11], without the addition of examinations or extension of a scanning session, which could be unpleasant for the subject. However, when images are analyzed numerically to extract meaningful data, as is done in radiomic studies, variations in acquisition and image reconstruction parameters can introduce changes that are not necessarily due to underlying biological effects [5]. Consequently, the used acquisition protocols should be highly standardized in order to collect a dataset that is suitable for reliable analyses avoiding issues that can create difficulties in comparing results. Radiomics can be performed using tomographic images from Computed Tomography (CT), Magnetic Resonance (MR) imaging and Positron Emission Tomography (PET) [12] studies. Each modality has its own imaging issues, which will be pointed out in the following subsections. Significant efforts are required to identify univocal acquisition and reconstruction protocols and to match them between scanners [13].

##### Computed Tomography (CT)

Relevant parameters have to be taken into account to obtain standardized protocols are related not only to image acquisition, but also to the reconstruction procedure, which delivers the image to the analysis step. Slice thickness, current (mA), and tube potential (kVp) are responsible for the photon statistics within a slice, therefore affecting the histogram and the noise level of an image. The axial field of view and the reconstruction matrix size change the voxel size within a slice, while the pitch, which is frequently optimized by each scan manufacturer, is crucial for the control of image noise. With respect to the reconstruction algorithm different approaches can lead to changes in Hounsfield units [13]. This may result in different quantitative features being extracted from the same acquisition protocol, with obvious negative effects on the reproducibility of radiomic analyses.

##### Positron Emission Tomography (PET)

Many issues related to the standardization of PET acquisition can be attributed to the calibration and quality controls are necessary to perform a quantitative examination [14]. With respect to CT, the scan and reconstruction parameters have to be standardized. For example, differences in grid size and post-reconstruction filter width imply large variations in the extracted features [15]. Additionally, the partial volume effect (PVE), which is mainly due to limited spatial resolutions and a relatively high noise contributions from PET systems, may quantitatively affect the voxel values and, consequently, the extracted features, especially in the case of small patterns that are comparable to the spatial resolution [16]. Consequently, there is a great interest in PVE correction methods that improve the reliability of quantitative index extraction [17]. Moreover, when dealing with PET quantitative acquisition, a precise patient protocol also has to be taken into account, with respect to dose calibration, blood glucose level, and uptake period [18].

##### Magnetic Resonance (MR)

Due to its complexity, MR can potentially play an important role in radiomic studies. MR images provide multi-parametric information, according to the used contrast weighting, which can be useful in categorizing a tumour. Ideally, MR images should all have, at least, the same field of view and acquisition matrix, field strength, and slice thickness, as these parameters strongly affect the Signal-to-Noise Ratio (SNR). Moreover, each acquisition technique requires specific expedients. For example, the commonly-used diffusion-weighted imaging (DWI) technique is strongly dependent on the k-space trajectory, gradient strength, and b-values, while dynamic contrast-enhanced (DCE) MR, and dynamic susceptibility contrast (DSC) MR depend on the contrast agent dose, administration procedure, and pulse sequence [13].

#### 2.1.2. Region of Interest Segmentation

This step requires a preliminary identification of target regions of prognostic value. Radiomics is based on the concept that restricting the features to mine at the input point may be inefficient, while it is worth capturing as much data as possible, and identifying the features with the highest prognostic values, only at the end-point of the process using database mining [5]. Consequently, sub volumes of interest within the lesions, representing physiologically-distinct volumes (habitats), can be captured and added to the analyses [19,20].

Once the volumes of interest have been identified, the segmentation strategy has to be chosen. This point is very critical as the resulting feature values depend on the adopted strategy and the segmentation accuracy. In radiomic studies, the preferred segmentation method should be time efficient and should also provide accurate and reproducible boundaries. Usually, manual segmentation by expert readers is considered the gold standard, but it is a time-consuming process with high inter-operator variability [21,22]. On the other hand, many automated methods have been developed across various image modalities and anatomical regions. They are completely reliable and reproducible in ideal conditions (such as normal structures and the absence of image artifacts), but they can fail in pathological situations, above all in complex cases of tumours with indistinct borders, because of inter- and intra-subject morphologic and contrast heterogeneity. Consequently, the best compromise has been identified in computer-aided detection systems [5,13,23] that work semi-automatically, with minimal user interaction (i.e., identification of seed points or manual correction). The use of semi-automated methods has also paved the way for three-dimensional (3D) segmentation. Volumetric segmentation allows a comprehensive view of the total tumour burden and volumetric assessment has shown good performance when related to different end points [24,25]. When comparing 3D with 2D approaches, it should be taken into account that tridimensional regions of interest, not only allow for a more complete description of the shape of the lesion [26], but also increase the number of points included in the statistical feature computation, leading to more reliable results, which do not suffer from sampling errors [27]. However, volumetric segmentation cannot be tackled with a standard, labor-intensive, manual approach. By reducing the manual workload, computer-aided approaches allow fast and reproducible 3D volumetric segmentations in large cohorts of patients, such as in radiomic studies [23].

#### 2.1.3. Descriptive Features

Once each ROI has been segmented, radiomic analyses are based on the automated extraction of features that robustly and quantitatively describe the attribute and the complexity of individual ROIs. These quantitative image features should offer information on the tumour phenotype and microenvironment, which represent an evolution of the semantic features, which have been introduced in the clinical evaluation of several oncological lesions (BI-RADS for breast cancer [28], PI-RADS for prostate cancer [29], VASARI for glioblastoma [30]). Semantic features are qualitative extracted by a radiologist that follows a controlled lexicon to describe lesions and represents a coarse attempt to approach radiomics. Their quantitative translation with mathematically-extracted descriptors, which go beyond of the expert eye of radiologist, can complement and overcome them with an operator-independent and high throughput approach.

Typically, extracted features can be divided into shape-based, as well as first-, second-, and higher-order statistical outputs.

##### Shape-Based Features

Shape-based features rely heavily on the segmentation approach used, and capture numeric information regarding geometric characteristics, such as size, shape, and spiculation. Some intuitive examples are:

- Volume:
  *V* = *N*∙*vs* (1)
  where *N* is the number of voxels within a segmented volume of interest, and *vs* is the voxel size of the acquisition.
- Surface area:
  (2) $$A=\;\overset{NT}{\underset{i=1}{\sum }}\frac{1}{2}\left|\vec{a_{i}b_{i}}\times \vec{a_{i}c_{i}}\right|$$
  NT is the number of triangles obtained from the triangulation of a tumour surface; $a_{i}$, $b_{i},$ and $c_{i}$ are the vertices of the *i*-th triangle. The surface area, together with the volume, and eventually, the maximum diameter, provide information on the size of a lesion.
- Compactness:
  (3) $$c=36\pi \frac{V^{2}}{A^{3}}$$
  This factor measures how much a lesion is different from a sphere, indicating, consequently, its irregularity.

##### First-Order Statistics

These features describe the distribution of voxel values without concern for their spatial relationships. They are generally histogram-based and can be used to quantify phenotypic traits [31]. Some examples are:

- Mean: shows the average intensity value and is given by:
  (4) $$\bar{X}=\;\frac{1}{N}\overset{N}{\underset{i=1}{\sum }}X\left(i\right)$$
  *X*(*i*) is the gray value of the *i*-th voxels within a region of interest. Other estimates of the central tendency, used in descriptive statistics, can be computed, such as the mode and median.
- Standard deviation: indicates how widely intensity values vary, and is computed as:
  (5) $$\sigma =\;\sqrt{\frac{1}{N-1}\overset{N}{\underset{i=1}{\sum }}{\left(X\left(i\right)-\bar{X}\right)}^{2}}$$
  Other measures of histogram dispersions are the variance and the mean absolute deviation. The variability within a volume can also be indicated by common statistics, such as minimum, maximum and range values.
- Entropy: a statistical measure of randomness within a data sample, given by:
  (6) $$entropy=\;-\overset{N_{l}}{\underset{i=1}{\sum }}P\left(i\right)log_{2}P\left(i\right)$$
  where *P* is the first-order histogram of the volume of interest, computed on N_l_bins. Additionally, uniformity and energy can be used to measure the randomness of a volume histogram.
- Skewness: a parameter that describes the asymmetry of a histogram around the mean, calculated as:
  (7) $$skew=\;\frac{\sum_{i=1}^{N}{\left(X\left(i\right)-\bar{X}\right)}^{3}}{N\sigma^{3}}$$
- Kurtosis: a parameter that depicts the degree of peakedness (broad or narrow) of a histogram and is given by:
  (8) $$kurt=\;\frac{\sum_{i=1}^{N}{\left(X\left(i\right)-\bar{X}\right)}^{4}}{N\sigma^{4}}$$

##### Second-Order Statistics

Second-order statistical descriptors, typically referred to as “texture” features, describe spatial relationships between voxels with similar gray levels within a lesion. They provide a measure of intralesional heterogeneity [32]. Typically-used techniques are the Gray Level Co-occurrence Matrix (GLCM) [33], also known as Haralick features, the Gray Level Run-Length Matrix (GLRLM) [34], and the Gray Tone Difference Matrix (GTDM) [35].

- Gray Level Co-occurrence Matrix: These matrices determine how often a pixel of intensity *i* finds itself within a certain relationship to another pixel of intensity *j*. A GLCM is a joint probability function, defined as P (*i*,*j*;*d*,*a*), where the elements (*i*,*j*) represent the number of times that intensity levels *i* and *j* occur in two voxels separated by distance *d* in the direction *a*. The matrix size depends on the intensity levels within a segmented lesion and the number of matrices on the chosen *d* and *a*. For each matrix, several features can be extracted, and the final value for each *d* considered is obtained as the mean of the feature over the directions. Examples of characteristics that are mineable from each matrix are: Mean, standard deviation, and entropy for the joint and marginal probabilities, autocorrelation, cluster prominence, cluster shade and tendency, contrast, correlation, difference entropy, dissimilarity, energy, homogeneity, etc. [36].
- Gray Level Run-Length Matrix: A gray level run is the number of consecutive pixels having the same grey levels. In a GLRLM, defined as p (*i*,*j*;*a*), the row indices represent the discretized gray values and the column indices are the number of consecutive occurrences of the *i*-th gray value in direction *a*. The matrix size, consequently, depends on the number of gray values in a lesion (number of rows) and the maximum run length (number of columns). A GLRLM can be obtained for each *a*, and the textural features can be obtained as the mean over the directions of the values extracted from each matrix. Examples of mineable features are: Short and long run emphasis, gray level non-uniformity, run-length non-uniformity, run percentage, low and high gray level run emphasis, etc. [30].
- Gray Tone Difference Matrix: A column matrix, in which elements s (*i*) are the sum over the set of pixels having gray tone *i*, of the difference between the voxels of the set and the mean value, computed over the corresponding neighborhood. Consequently, the matrix depends on the size of the neighborhood. From GTDM, several features can be computed: Coarseness, contrast, busyness, complexity, and strength.

##### Higher-Order Statistics

Higher-order statistics impose filter grids on an image to extract repetitive or non-repetitive patterns. From filtered images, first- or second-order features are computed. Filters that are most often used in radiomic studies are:

- Laplacian of Gaussian filter [32]: This allows the highlighting of structures at a particular scale, corresponding to the width of a filter. Consequently, increasingly coarse texture patterns can be extracted from an image and analyzed using second-order statistics.
- Gabor filters: These allow for edge detection in different directions and widths [37]. For each filtered image, the Gabor energy feature can be extracted as a sum of the square intensity over all tumour pixels.
- Wavelet transform: This decouples textural information by decomposing the input image into low- and high-frequency coefficients without losing spatial localization. In particular, high-frequency coefficients also contain information on texture directionality. If an undecimated scheme is chosen, lesion segmentation, identified in the original image, can be used for computation of the first-order statistics and textural features from the wavelet coefficients.
- Fractal dimensions: These are estimates of object complexity. Fractal dimensions describe the relationship between the change in a measuring scale and the measurement at that scale [31], and can be calculated using a 3D box-counting algorithm [15]. Successively, values, such as mean and standard deviation, can be extracted.

As a result of features extraction, up to hundreds of features will be delivered to the statistical analysis, for only the radiomic part.

### 2.2. Genomics

**Figure 2:**
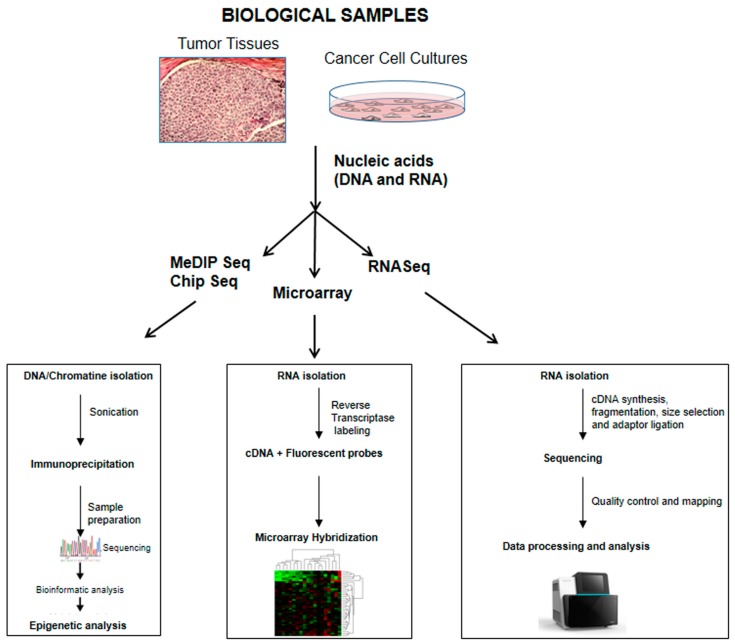
Schematic workflow of the most used genomic approaches that could be applied in the field of radiogenomics. Methylated DNA immune precipitation sequencing (MeDIP Seq) and Chip sequencing (Chip Seq) provide information about DNA methylation, DNA/protein interactions, and histone modification. Microarray is a technique used to measure the expression levels of large numbers of genes. RNA sequencing (RNA Seq) allows performing an in-depth analysis of the transcriptome with the identification of novel transcripts, alternative splicing allele specific expression, gene fusions, and genetic variations. Moreover, RNA Seq can give information about the transcriptome dynamics such as RNA editing, small insertions/deletions, exon connections, non-coding RNAs, and small RNAs.

Genomics is an interdisciplinary field of science focusing on genomes, which is actually performed using a combination of high-throughput molecular biology technologies with complex computing and math techniques (bioinformatics analysis). Nowadays, technological improvements have provided the possibility of determining the expression of thousands of genes involved in different fields of human health and pathologies. Generally, two technologies are critical for genomics analyses: (1) microarray; and (2) next-generation sequencing (NGS) including DNA and RNA sequencing (Figure 2).

#### 2.2.1. Microarray

Microarray technology allows the understanding of complex functional mechanisms that are involved in physiological and pathological cellular processes. Indeed, DNA microarrays provide a rapid and accurate analysis of global gene expression, in contrast to previous methods for quantifying mRNAs, such as Northern blotting or quantitative PCR, that are able to measure a few genes at a time. DNA microarrays method, individual PCR-amplified double-stranded cDNA fragments (about 500 base pairs) are spotted onto microscopic glass slides; typically this technique exploits a spotted two-color hybridization to visualize and measure gene expression levels when comparing gene expression profiles from multiple samples [38,39]. In case of comparisons across a large number of samples, a common reference sample is usually used as standard in all experiments [40,41,42]. The result of a DNA microarray is like a snapshot of actively expressed genes and transcripts (transcriptome) at a given point in time. However, although microarrays are used for identifying the expression of known genes and transcripts, this method fails to detect unidentified genes or transcripts. The solution to this issue can be circumvented by the use of in-depth sequencing analyses, such as Next-generation sequencing (NGS) analyses, discussed below. DNA microarrays are also exploited in the field of epigenetics, which refers to changes in gene expression occurring without alterations to the DNA sequence. It is known that many neoplastic transformation events are connected to deregulated epigenetic machinery, such as DNA methylation of the CpG island within promoter regions and the covalent modification of histone proteins, such as acetylation, phosphorylation, methylation, and ubiquitilation, are able to regulate gene activity [43]. For large sample size genome-wide DNA methylation studies, the most used platform is the Infinium HumanMethylation450 BeadChip kit (Illumina, San Diego, CA, USA). This technology is able to obtain a rapid quantitative DNA methylation analysis of more than 485,000 CpG dinucleotides (methylation sites) located across the genome [44] (Figure 2). As reported in the Discussion section, most radiogenomics studies, on a variety of human cancers, used microarray technology. In these studies, genomic data were compared using different radiomic approaches such as MRI, CT, and PET. The aim of these studies was to identify imaging and genomic features that are able to ameliorate the diagnosis and prognosis of cancer patients.

#### 2.2.2. Next-Generation Sequencing

Next-generation sequencing (NGS), also known as high-throughput sequencing, comprises a number of different modern technologies that are able to provide more accurate information than the previously-used Sanger method, revolutionizing the study of genomes and representing new opportunities for clinical applications [45,46]. The NGS-era started in 2004 with the introduction of massively parallel sequencing platforms [47] (e.g., the Applied Biosystems and the Helicos BioScience HeliScope) able to produce 400 million of 25–35 bp reads [48]. Although there are different technical details, a single DNA molecule in each sample is, first, fragmented, amplified, and then sequenced. A common feature among NGS technologies is that the template is attached to a solid surface or support, which enables, simultaneously, thousands, and up to billions of sequencing reactions. The massive amount of data generated is attractive, and the elimination of the cloning step for the DNA fragments to be sequenced is the greatest benefit of these new technologies. The broadest application of NGS may be the resequencing of different genomes and, in particular, human genomes for an in-depth understanding of genetic differences in health and disease. Furthermore, these platforms have been used in many genomic applications, leading to a wide range of so-called “Seq” protocols, such as RNA-Seq for transcriptomics, Chip-Seq for DNA-protein interaction analyses, and methyl-Seq for epigenetic mark profiling. Several programs can be used for data handling and visualization, quality assessment, interpretation, and statistical analyses [49,50,51] (Figure 2).

##### RNA-Sequencing

Particular interest in oncology is focused on the field of transcriptomics for the identification and quantification of the RNA in cells, tissues, or biological fluids, representing a powerful tool for the assessment of specific biological activities [52]. In a single RNA-Seq experiment, it is possible to investigate, not only gene expression, but also alternative splicing [53], novel transcripts [54,55], allele specific expression [56], gene fusions [57], and genetic variations [58,59]. Moreover, RNA-Seq can provide more interesting information regarding transcriptome dynamics, such as RNA editing, small insertions/deletions, exon connections, non-coding RNAs, and small RNAs [60]. Today, there are three widely-accepted, commercially-available NGS devices for RNA-Seq: 454 GS FLX (up to 400 bp) (Roche, Basel, Switzerland), Genome Analyzer II, with paired-end reads up to 100 bp (Illumina, San Diego, CA, USA), and SOLiD (up to 35–50 bp) (Applied Biosystems, Foster City, CA, USA) [61,62]. Although each platform works differently, they are all based on similar principles: Shearing target nucleic acids into small pieces, binding individual molecules to a solid surface, amplifying each molecule into a cluster, copying one base at a time, and detecting different signals for each nucleotide base. The majority of the platforms only allow for the sequencing of DNA molecules. Therefore, RNA molecules are, first, reverse transcribed into cDNA. Once reverse-transcription is complete, the RNA molecule is removed. At the end of the entire process, the result is a sequence of images, where each lighted spot corresponds to a cluster and the color of each cluster represents a different base type [51].

The first step of RNA-Seq data analysis is the quality control of the raw reads, particularly the determination of sequence quality, GC content, overrepresented k-mers, and duplicated reads, in order to detect sequencing errors, PCR artifacts, or contamination [63]. An important indicator of sequencing quality/accuracy and absence of contaminating DNA is represented by the percentage of mapped reads [64,65]. After quality control, NGS-data analysis is performed by mapping the sequence reads. Indeed, reads are aligned to a reference genome, or to reference transcripts, or assembled de novo without a referenced genomic sequence to produce a genome-scale transcription map, consisting of the transcriptional structure and or the expression level for each gene. When a reference genome is available, RNA-Seq analysis involves the mapping of the reads onto the reference genome or transcriptome, even with the limitation of discovery new transcripts. If an organism does not have a sequenced genome, a de novo assembling approach to produce a genome-scale transcriptional map is necessary [66,67,68,69]. For the identification of novel transcripts, several software packages and algorithms are used to assess splice junctions and transcription start and end sites [70,71,72,73,74,75]. Gene-finding prediction tools, such as Augustus [76], can exploit RNA-Seq data to better annotate protein-coding transcripts [77].

The second phase of RNA-Seq analysis provides the quantification of transcript expressions using programs such as HTSeq-count [78] or feature Counts [79], based on the aggregation of the raw counts of mapped reads. Quantitative gene expression data from RNA-Seq have been shown to be comparable to those of microarrays, but with a better dynamic range and lower detection limit for low-expressed transcripts [59] (Figure 2).

RNA-Seq is also used to study the biological role and signature of small RNAs (sRNAs). Although sRNA-Seq libraries are rarely sequenced as deeply as classical RNA-Seq libraries, and bioinformatics analysis is different from standard RNA-Seq protocols, obtained sRNA reads are aligned to a genome or transcriptome reference using bioinformatic tools, such as Bowtie2 [80], STAR [81], or Burrows-Wheeler Aligner (BWA) [82].

Innovations in RNA-Seq have made quantitative transcriptome analysis of a single cell possible, even when RNA-Seq is performed on a large number of cells in the same run [83]. Furthermore, methods that integrate DNA whole exome sequencing (DNA-WES), or Chip-Seq, with RNA-Seq have allowed increased mutation detection performance [49,84,85]. Finally, an in situ method of RNA-Seq has also been developed for preserved tissue sections or cell samples [86].

RNA-Seq offers several advantages compared with other transcriptomics methods [59,87], providing high-throughput solutions for the construction of single-base resolution expression profiles with low background noise and a low amount of required starting RNA. Furthermore, it can generate millions of reads in a single run. Nevertheless, few studies used the RNA-Seq technology in combination with radiomic technologies (MRI) to address clinical issue, are reported in Table 1. Indeed, although RNA-Seq provides results that are superior to microarray analysis, in terms of sensitivity, specificity, and abundance estimation, microarrays are still used more than RNA-Seq. This is probably due to costs, run time, and the large volume of data, that make it necessary to dedicate platforms to data storage (Big Data). In light of this, although RNA-Seq is promising, technological improvements for reducing costs, improving data processing/storage, and gold standards for analyses are necessary to best use this powerful platform in research laboratories and clinics.

#### 2.2.3. Immunohistochemistry

The utility of the immunohistochemistry (IHC) technique for the improvement of microscopic diagnosis of neoplasia is known. This method aims to characterize cellular or tissue constituents for example by identifying therapeutic targets (markers) for cancer by taking advantage of antigen-antibodies interactions. This method expects different steps: Deparaffinization of tissue sections, quenching of endogenous enzymes to avoid false positive results, antigen retrieval, blocking of nonspecific binding sites, binding primary antibodies, binding with biotinylated secondary antibodies, detection by using one or several methods as peroxidases- antiperoxidase, avidin biotin conjugates, peroxidase complexes, or by using a polymer-labelling two-step method, addition of chromogen substrate, counterstaining, dehydrating, and cover slipping the slide. Advantages of these methods include protein localization and distribution, applicable for different sizes of tissue biopsies, and fixed tissues and validation of other high-throughput studies (DNA microarray). Nevertheless, these methods show some limited ability regarding the detection of protein modifications and quantifications [88].

### 2.3. Radiogenomic Data Analysis

Once both radiomic and genomic features are extracted for each subject, radiogenomic analysis are performed on a population dataset, including the clinical outcome related to each sample. A prediction study is aimed to forecast either an overall or progression-free survival or response to a particular treatment [89]. In addition, prediction studies are needed to recognize a class of disease or to predict genotype from imaging phenotype and vice-versa [90]. A simple radiogenomic correlation study, by contrast, can be performed investigating the mutual statistical relationship between radiomic and genomic features for a given disease, regardless the clinical outcome [91].

Considering the very large dimensionality of the feature space, especially when dealing with multimodal imaging, advanced algorithms that rank features by their importance for a given disease outcome are often essential to reduce over fitting, increase reliability, and address the curse of dimensionality [92]. The latter, which refers to a crucial aspect in radiogenomic data analysis, can be roughly summarized as the requirement of higher sample sizes as the number of the features increase.

Feature selection can be performed as a preparatory step (filtering approach) or can be embedded within the classification or regression procedures, identifying a sparse set of features that can be used to train highly-accurate predictors of individualized outcomes. In References [93,94] features were ranked and selected on a test-retest reproducibility basis before proceeding with a correlation analysis; redundancy was avoided by first eliminating highly-correlated features [95]; see Reference [96] for a comprehensive description and evaluation of the most popular feature filtering methods. As an instance of embedded feature selection, the work in Reference [97] employed a LASSO regularization [98] within a logistic regression to assess the predictive power of each feature.

Since the domain of each feature can be different, data normalization strategies should be considered on the basis of statistical analysis methods; a well-established choice is the use of a z-score transformation [96], but, as previously demonstrated [36], radiomic data can be employed without any normalization processing.

Once each sample is arranged into a suitable feature space, data can be classified in a supervised or unsupervised manner. Unsupervised methods, such as clustering and principal component analysis, are data-driven methods aimed at discovering clusters, representing different radiogenomic phenotypes, within the feature space [99]. Supervised methods, such as machine learning approaches, involves a prior labelling of data containing the clinical outcome (e.g., overall survive/not survive; responder/non-responder) and a training step to infer a model underlying the data, which can be used for predicting the labels of unseen observations. Classifiers, such as support vector machines (SVM), span different areas of computer and information science; see Reference [96] for a description of well-established methods employed in radiogenomics. Alongside typical machine learning methods, the family of deep learning algorithms is gaining increasing interest as robust classifiers for the radiogenomic domain [100,101]. It is worth mentioning that machine learning and correlation analyses do not solve the problem of causal inference in observational data sets [4]. Therefore, conventional radiogenomic methods carry out a correlative association of radiomic signatures with gene signatures. Alternatively, a recent study [102] proposed a preclinical radiogenomic methodology to demonstrate that image features are causally related to genetic factors.

**Figure 3:**
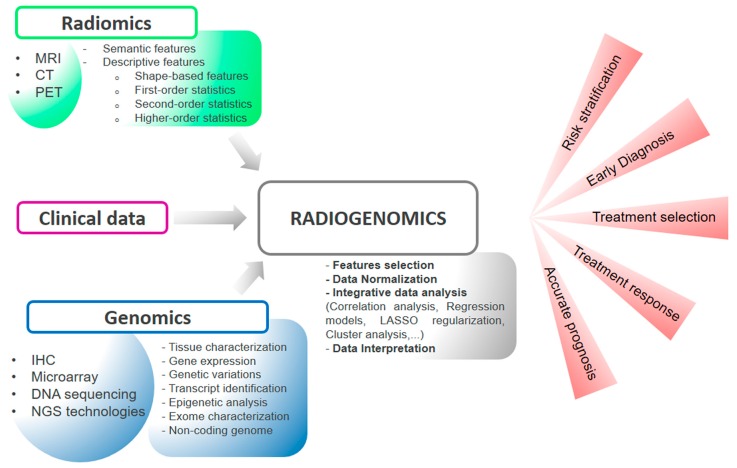
The figure shows a general workflow for radiogenomic study. The first step includes data acquisition (clinical information, imaging and genomic data). Subsequently, data are normalized and underwent an integrative analysis to characterize each radiomic feature and identify specific underlying molecular functions. The overall flow, here schematically depicted, could represent a novel integrated approach for cancer diagnosis and prognosis.

Figure 3 shows the whole radiogenomics pipeline, in light of methodological issues described throughout this section.

## 3. Discussion

In the last five years there has been a significant increase of studies that use the radiogenomic approach to obtain clinically-significant information to be translated into the clinical practice. As outlined in Figure 3, there are multiplicity of methods involved in radiogenomics, as well as wide variety of the possible outcome of a radiogenomic study.

Table 1 summarizes most of the relevant oncological studies in radiogenomics, crossing imaging and biological data. We searched multiple electronic databases for original research studies including the following words: “radiogenomics and breast cancer”, “radiogenomics and glioblastoma”, “radiogenomics and lung cancer”, “radiogenomics and kidney cancer”, “radiogenomics and renal cancer”, “radiogenomics and hepatic carcinoma” “radiogenomics and prostate cancer”. We excluded studies that associated the term radiogenomics to the radiation therapy response. The studies are classified according to the neoplastic disease and for each of them the methodological approach, as introduced in the previous sections, and the performed statistical analysis are pointed. In the next sections, the main results of the application of the radiogenomic approach as well as the techniques employed will be deeply discussed for each oncological disease.

### 3.1. Radiogenomics in Breast Cancer

The election imaging method for Breast Cancer (BC) was MRI (78%) [91,97,103,104,105,106,107,108,109,110,111,112,113,114,115,116,120,121]. About 78% of the BC published papers were correlation studies [91,103,104,105,106,107,108,109,110,111,112,114,117,118,119,120,122,123] and the most used genomic data was IHC results (65%) [103,104,105,107,108,111,112,113,114,115,116,117,118,119,120]. However, although IHC is routinely applied in the clinical practice, it is not an “omic” approach, remaining the elective method to determine the BC molecular subtypes (Luminal A, Luminal B, basal-like, and triple-negative (TNBC)), useful for therapeutic indications [154].

The first study, dates back to 2009, in which authors found a significant correlation between high histological grade, unifocal lesion, mass lesion type, smooth mass margin, rim enhancement, persistent enhancement pattern, and very high intratumoural signal intensity on T2W MR images with the triple-negative subtype [108]. This finding was confirmed by Sung and colleagues [105]. Cipolla et al. and Molinari et al. reported a significant correlation between ADC values with tumour aggressiveness in terms of grade and the Ki67proliferation index [111,112]. In addition, the Ki67 index and ER score correlated with the roundness of tumour, although in an independent manner [114]. Only three studies correlated IHC results and PET/CT imaging features, reporting an association between FDG uptake and Ki67 [117,119], FDG uptake and tumour size in TNBC [117] and higher SUVmax with triple-negative and HER2-positive tumours [118].Three works focused on prediction studies combining IHC with MRI [113,115,116] or with PET [116].

Few works focused on correlation (21%) and predictive (12.5%) studies performed by the use of genomic data (DNA microarray or RNA Seq) in combination with MRI [91,97,106,109,110,121] and PET features [122,123]. According to the primary goal of radiogenomics, as described in previous sections, these studies clearly fulfill the radiogenomics concept. Specifically, Yamamoto et al. evidenced that contrast-enhanced MRI systematically correlated with complex transcriptome profiles [109] paving the way for the generation of imaging biomarkers correlated with individual patient biology. Recently, Sutton et al. evidenced in patients with invasive ductal carcinoma that MR imaging-derived features were correlated with the Oncotype Dx RS scoresbased on the expression levels of 21 BC-associated genes [106]. The ODx score correlated with the magnitude of chemotherapy, as well as the 10 years risk of distance metastases, representing a possible prognostic and predictive surrogate genomic-based test. The usefulness of in-depth imaging analysis of BC vascularity by MRI, using the Dynamic Contrast-Enhanced (DCE) parameters, has been witnessed in a recent study by Mehta et al. [121]. The authors found that DCE MRI could provide criteria for patient stratification in the case of an anti-angiogenesis trial. Indeed, patients with a high Ktrans value at diagnosis disclosed a higher complexity in terms of proliferating cancer genes, as well as increased vasculature. Based on these findings the authors postulated that DCE-MRI could evidence, in a non-invasive way, candidates that could benefit from anti-angiogenic therapy. Future studies are needed to evaluate, in large BC patient cohort, the clinical usefulness of the MRI-derived imaging biomarkers. In this context it is important to consider a recent paper where, for 91 breast-invasive carcinomas, it was possible to integrate The Cancer Genome Atlas (TCGA) data with the MRI data from The Cancer Imaging Archive (TCIA) [91]. In this work quantitative MRI phenotypes, such as tumour size, shape, margin, and blood flow kinetics, were associated with their corresponding molecular profile, including DNA mutation, gene, miRNA, and protein expression, as well as copy number variation. Only two studies used RNA-Seq methods in combination with MRI features [97,110]. The first one, a predictive study, analyzed the genomic and radiomic data of 91 BC patients from TCGA and TCIA in order to predict clinical outcomes [97]. The work of Yamamoto, instead, was a correlation study which introduced, for the first time, the use of long non-coding RNA (lncRNA) as genomic data combined with MRI features. A radiogenomic analysis allowed identifying eight lncRNAs, three of which were unnamed. The pathway analysis showed that known lncRNAs molecules were involved in metastasis, cell cycle, cell death and survival, cellular development, and cellular growth and proliferation. In particular, higher levels of lncRNAs HOTAIR and LINC00511 were found in tumour samples of invasive ductal carcinoma than in normal breast tissue with a significant positive correlation with texture features [110].

### 3.2. Radiogenomics in Glioblastoma Multiforme

From Table 1, the wide use of semantic features in GBM appears obvious; these are preferred to descriptive features, more suitable for radiomic analyses, due to their high level of standardization. It is worth to mention that the majority of GMB radiogenomic study exploits the intrinsic multimodality of MR, considering both anatomical and functional features. Therefore, the radiogenomic approach has proven successful in determining the MRI phenotype associated with genetic alterations detected via DNA microarray analysis in GBM. The first study dates back to 2007. In this study, the authors evaluated Vascular Endothelial Growth Factor (VEGF) and related gene expression in 71 malignant GBM tissues in order to analyze their relationship with edema and survival. By using DNA microarray and MRI methods, they found that VEGF expression was predictive of survival with tumours with little or no edema in GBM-affected patients [136]. Subsequently, Diehn and colleagues combined neuroimaging (MRI) and DNA microarray analysis in a correlation/predictive study to create a multidimensional map of gene expression patterns, which provided clinically relevant insights into tumour biology. This study offered a potential strategy for noninvasively selecting patients who may be candidates for individualized therapies [124]. In the same year, in a similar study, the relationship between gene expression and MRI enhancement in GBM was evaluated. Results showed that gene expression was able to discriminate magnetic resonance imaging features in incompletely enhancing (IE) and completely enhancing (CE) tumours and to predict patient survival [134]. In order to increase the use of non-invasive imaging as an emerging field of treatment response and personalized medicine, over the years, other works have been published in the field of the radiogenomics for the study of brain tumours, through the analysis of gene expression and DNA copy number variations in tumour tissues. Most of these are correlation studies that were performed using DNA microarray and MRI imaging modalities [125,126,129,131,132,133,139,141]. On the other hand, a plethora of studies investigated the possible predictive value of the radiogenomics approach, showing promising results [127,128,130,135,137,140,143]. The only study that used the CT modality associated with a DNA microarray assay showed a correlation between anti- and pro-angiogenic genes with tumour perfusion parameters [138]. In addition, recently, Demerath and colleagues used MRI and the less innovative molecular technique of IHC, to focus their study on the use of mesoscopic measures as surrogate markers for specific gene expression patterns in GBM [142].

### 3.3. Radiogenomics in Lung Cancer

Most radiogenomics works applied to lung cancer are predictive study. One of the first works, dating back to 2012, was focused on CT and PET images features in patients with non-small cell lung cancer (NSCLC) [145]. The authors explored the relationship between differential genome-wide expression using DNA microarrays and different FDG uptake levels in NSCLC, finding a gene expression signature associated with prognostically-relevant FDG uptake features. In particular, they exploited public gene expression microarray data and 180 image features from CT and PET/CT, identifying 243 statistically-significant pairwise correlations between image features and metagenes to assess prognosis and therapeutic response [145]. Subsequently, in a large cohort of patients with diagnosed NSCLC, Nair and colleagues found that Nuclear factor-κB (NF-κB) IHC expression was related to tumour metabolism, measured using FDG-PET, and prognosis provided a methodology for studying tumour biology using computational approaches [146]. Four hundred and forty quantitative image features, describing tumour phenotype characteristics (tumour image intensity, shape, texture and multiscale wavelet), were defined, and Aerts et al. compared these radiomic signatures with gene-expression profiles using gene-set enrichment analysis (GSEA) [36]. They found that a large number of radiomic features had prognostic power in lung and head-and-neck cancer patients, many of which were not previously identified as significant. Indeed, this radiogenomic approach revealed that a prognostic radiomic signature, capturing intratumour heterogeneity, was associated with gene-expression patterns, providing an opportunity to improve decision-support in cancer treatment at low cost. Recently, whether epidermal growth factor receptor (EGFR) and KRAS mutation status can be predicted using CT imaging data, as been investigated [144]. In this study, the authors found a statistically-significant model for predicting EGFR, but not for KRAS mutations, showing the potential of quantitative imaging to predict molecular properties in a non-invasive manner.

### 3.4. Radiogenomics in Kidney Cancer

Few works using radiogenomic models have been published in the field of kidney carcinoma since 2014. These preliminary studies revealed the association between CT features of clear-cell renal cell carcinoma (ccRCC) with somatic mutations in several genes detected by PCR amplification and DNA sequencing. These genes (*VHL*, *PBRMI*, *SETD2*, *KDM5C*, *BAP1* and *MUC4*) were known to be related to advanced grade, stage, and reduced survival prognosis [147,148]. Study published by Karlo et al. was upgraded by the work of Jamishidi et al., were the authors constructed a complex, multi-feature imaging predictor by using a multi-gene predictive gene expression signature and 28 CT images [148].Through these discoveries, the authors identified imaging features that were potentially predictive of outcomes.

### 3.5. Radiogenomics in Prostate Cancer

Several studies have addressed the utility of MRI to determine the aggressiveness of prostate cancer using ADC, DCE, and Gleason score parameters. To our knowledge, the first studies in prostate cancer, investigating the associations between MRI parameters with the genomic markers, were published in 2016 [152,153]. McCann and coauthors studied the correlation between quantitative imaging features of multi-parametric MRI and phosphatase and tensin homolog (PTEN) protein expression by IHC analysis. They found a significant, although weak, associations between the extracellular space and plasma (Kep) and the Gleason score with PTEN expression, claiming that the use of this model can improve the risk assessment of patients with prostate cancer. The study performed by Stoyanova et al. showed a correlation between radiogenomic parameters and prostate cancer, exploiting MRI-guided biopsy. They identified radiomic features in normal-appearing tissues associated with high-risk gene expression profiles, as well as radiomic biomarkers in cancer tissues associated with genes of adverse outcomes. Gene expression profiles of tissue specimens were determined using DNA microarray.

### 3.6. Radiogenomics in Liver Cancer

By using an integrated imaging-genomic approach, the first study on liver cancer dates back to 2007. The aim of that work was to determine whether conventional contrast-enhanced CT could be used in identifying imaging phenotypes associated with a doxorubicin drug response gene expression program in hepatocellular carcinoma (HCC) [94]. They found that tumour margins in arterial phase images showed significant correlation with (i) the doxorubicin-response gene expression program; (ii) HCC venous invasion; and (iii) tumour stage. Furthermore, tumours with higher tumour margin scores were associated with the doxorubicin resistance transcriptional program, and had a greater prevalence of venous invasion a worse stage. In the same year, a study was published showing that 28 dynamic imaging traits in CT correlated with gene expression programs of primary human liver cancer. Specifically, the combinations of imaging features were able to reconstruct 78% of the global gene expression profiles, revealing cell proliferation, liver synthetic function, and patient prognosis [150]. Recently, Miura and coauthors aimed at evaluating the clinicopathological and biological properties of high HCC-correlating ethoxybenzyl-magnetic resonance imaging hyperintensity with gene expression signatures [151]. They identified 53 up-regulated and 71 down-regulated probe sets in the high-HCC group compared with the low-HCC group, showing that clinicopathological and global gene expression analyses revealed low-grade malignancies in high HCCs compared with low HCCs.

### 3.7. General Considerations

Imaging features listed in Table 1 show that the majority of studies are based on a qualitative and supervised analyses of imaging data, therefore, partially exploiting the full potential of radiomics features as described above. Although the NGS approaches could offer high capabilities for in-depth characterization of tumour biology, most of the radiogenomic studies integrate genomic features by using microarray to assess differential changes in gene expression levels. In fact, the application of this methodology in radiogenomic studies is limited due to the lack of standard references for analysis, it is expensive in terms of cost and time, and Big Data are produced, which require a dedicate storage platform [155]. In addition, Table 1 shows a wide heterogeneity of methods for features generation, as well as the statistical analyses employed, supporting that radiogenomics is still an elusive concept, wherein the standardization of procedures should be addressed.

In this context, an interesting scientific debate begun about the potential utility of radiogenomics compared to separated imaging and genomics approaches in clinical practice [8,9]. One advantage of the integrated approach arises from the limitations of currently available datasets, exploiting its ability to deal with limited and incomplete data to generate meaningful information [8]. Scientific studies regarding the relationship between a few imaging features and a restricted number of gene expressions should not be considered as a pure radiogenomics approach. Radiogenomic studies must combine a large number of quantitative imaging features with a massive genomic signature using computer algorithms. In addition, both radiomics and genomics are needful for the clinical decision making and neither one can replace the other, but their potential can be increased through the interpretation of the two methods to improve the management of cancer patients. Furthermore, the study of mutual relationships between imaging and genomics can provide novel insights for the understanding of neoplastic transformation.

The large number of measured variables can lead to a “fishing expedition”; as discussed in Reference [81], such a poor practice can be avoided with appropriate prior hypotheses about the study. From a statistical point of view, multiple comparisons should be controlled by correcting the accepted criteria for significance, such as the well-known Bonferroni P value correction or the False Discovery Rate (FDR) [118]. In order to translate radiogenomic research to the clinical practice, reproducibility and reliability of the measures and procedures are crucial issues to take into account, requiring the validation on an independent test-retest data set. In addition, a large cohort study gives power to the method validation but, to date, in the published studies an acceptable sample size is lacking. Although genomics has been demonstrated to be a robust analytical tool, the performance of radiomic measurements is still under investigation. Parmaret et al. have investigated the reproducibility of image segmentation and subsequent radiomic feature extraction, showing the better performance of semi-automated techniques [23]. In a recent study Zhao et al. [119] showed that radiomic features are reproducible over a wide range of imaging settings, but they suggest awareness of the importance of properly setting imaging acquisition parameters.

## 4. Conclusions

As tissue imaging can correlate with tissue pathology, radiomics aims at creating imaging biomarkers that can identify the genomics of a disease, especially cancer, without the need for a biopsy. Integration with genomic data in the last twenty years, including DNA microarrays and RNA-Seq, as allowed the investigation of the relationship between cellular genomics and tissue-scale imaging. The simultaneous use of these apparently different methods to answer a clinical question is called radiogenomics, and is aimed at correlating cancer imaging and gene features.

The increasing amount of imaging and genomic data identified in several types of cancer is encouraging an integrated approach that combines phenotype and genetics for advancing tumour characterization and precision medicine. To this aim, radiogenomics represents a powerful strategy, potentially enabling clinical decision tools to enhance diagnostic accuracy, as well as to measure the response to drug or radiation therapy, leading to an overall improvement of patient management. However, as with all new diagnostic approaches, it is still in the early phases, limiting its ability to be used to improve cancer patient management requiring standardization of imaging and genomic protocols, image acquisition, and post-processing. The next challenge of radiogenomics will be to identify specific signatures of intra- and inter-tumour heterogeneity in a proper anatomic context. In this way, the radiogenomic analysis of cancer heterogeneity could offer validity by adding and aiding in the assessment of tumour aggressiveness in a variety of clinical settings and oncological outcomes.

## Figures and Tables

**Table 1:** Radiogenomic studies published in oncology.

Tumour type	Rationale of the Study	Number of sample	Imaging data	Imaging Features	Segmentation	Genomic Features	Statistical Analysis	Ref.
BREAST CANCER (BC)	Correlation	275	MRI (T1WI, T2WI, DCE)	Shape-based features, second- and higher-order statistics, kinetic parameters	Semi-automated	IHC	Binary multivariate logistic regression model and univariate models	[103]
	Prediction	91	MRI (DCE)	Shape-based features, second-order statistics, kinetic parameters	Semi-automated	RNA Seq Microarray (TCGA)	Logistic regression with LASSO regularization and ROC analysis	[97]
	Correlation	48	MRI (T1WI, T2WI, DCE)	Shape-based features, second-order statistics, kinetic parameters	Semi-automated	IHC	Multivariate logistic regression models	[104]
	Correlation	221	MRI (T1WI, T2WI, DCE)	Semantic features	Manual	IHC	Wilcoxon test and Fisher’s tests	[105]
	Correlation Prediction	95	MRI (T1WI, T2WI, DCE)	Shape-based features, first- and second-order statistics	Manual	IHC Microarray	Multiple linear regression analysis and Spearman’s rank correlation	[106]
	Correlation	178	MRI (T1WI, T2WI, DCE)	Shape-based features, first- and second-order statistics	Manual	IHC	Multiclass support vector machines with the a leave-one-out cross-validation approach	[107]
	Correlation	176	MRI (T2WI DCE)	Semantic features	Manual	IHC	Chi-squared and Fisher’s tests	[108]
	Correlation	353	MRI (T1WI, DCE)	Semantic features	Manual	Microarray	Spearman rank-correlation	[109]
	Correlation	109	MRI (T1WI, DCE)	Shape-based features, first- and second-order statistics, kinetic parameters	Automated	RNA Seq	Cox regression analysis	[110]
	Correlation	92	MRI (T2WI, DWI, DCE)	Semantic features, ADC	Manual	IHC	Mann–Whitney U and Kruskal–Wallis H tests	[111]
	Correlation	115	MRI (T1WI, T2WI, DWI, DCE)	ADC	Manual	IHC	Mann–Whitney U and Kruskal–Wallis H tests	[112]
	Prediction	50	MRI (T1WI, T2WI, DCE)	Semantic features	Manual	IHC	Student’s unpaired *t*-test, one-way ANOVA, Chi-squared and Fisher’s test	[113]
	Correlation	282	MRI (T1WI, T2WI, DCE)	Shape-based features	Manual	IHC	Multiple linear regression analysis	[114]
	Prediction	96	MRI (T1WI, T2WI, DWI, DCE)	Shape-based features, ADC	Manual	IHC	Multivariate logistic regression analysis	[115]
	Prediction	214	MRI (T1WI, T2WI, DWI, DCE) PET/CT	ADC, SUV	Manual	IHC	Mann–Whitney *U* and Kruskal–Wallis *H* tests	[116]
	Correlation	103	PET/CT	SUV	Manual	IHC	Chi-squared test, Fisher’s and Wilcoxon tests	[117]
	Correlation	552	PET/CT	SUV	Manual	IHC	Univariate and multiple linear regression analysis	[118]
	Correlation	82	PET/CT	SUV	Manual	IHC	Chi-squared test, Fisher’s and Mann Whitney tests	[119]
	Correlation	91	MRI (DCE)	Shape-based features, second-order statistics, kinetic parameters	Semi-automated	Microarray (TGCA)	Regression and clustering analysis	[91]
	Correlation	228	MRI (T2WI, DCE)	Kinetic parameters	Semi-automated	IHC	Kruskal–Wallis *H* test	[120]
	Prediction	36	MRI (DCE)	Kinetic parameters	Manual	IHC Microarray	Wilcoxon test, Spearman’s rank correlation, and Kruskal–Wallis *H* test	[121]
	Correlation	36	PET	SUV	Manual	IHC Microarray	Two-way unsupervised hierarchic clustering and Spearman’s rank correlation	[122]
	Correlation	18	PET	SUV	Manual	Microarray	Rank-rank hypergeometric overlap	[123]
GLIOBLASTOMA (GBM)	Correlation Prediction	25	MRI	Semantic features	Manual	Microarray	Correlation analysis	[124]
	Correlation	78	MRI-FLAIR, T1-c	Size, volume	Automated	TCGA	Pathways genomic analysis	[125]
	Correlation	23	MRI (T1-c, DSC)	Semantic features	Manual	Microarray (GSEA)	Correlation analysis	[126]
	Correlation Prediction	76	MRI (TCIA) MRI (T1-c, FLAIR)	Semantic features	Semi-automated	Microarray (TCGA)	Student’s *t*-test ,ROC AUC analysis	[127]
	Correlation Prediction	92	MRI (TCIA)	Semantic features	Manual	Microarray (TCGA)	Hierarchical clustering and survival analysis	[128]
	Correlation	48	MRI anatomical	Second-order statistics	NA	CGH array exome sequencing	Multivariate predictive decision-tree models	[129]
	Correlation Prediction	55	MRI (TCIA)	Semantic features	Manual	Microarray (TCGA)	Cox proportional hazards modeling and correlation analysis	[130]
	Correlation	21	MRI (DSC)	Mean values	Manual	Microarray	Cox regression analysis	[131]
	Correlation	152	MRI (DWI,DSC, SWI,T1WI,T2W2)	First-order statistics, Semantic features	Manual	Microarray	Hierarchical clustering	[132]
	Correlation	13	MRI (DWI, DSC)	Mean values	Manual	Microarray	Correlation analysis	[133]
	Correlation Prediction	52	MRI (T1-c,DSC)	Clinical scores	NA	Microarray	Univariate Cox proportional hazard models	[134]
	Prediction	78	MRI (T1-c, DSC)	Semantic features	Manual	Microarray (TGCA)	Proportional Hazards Model	[135]
	Prediction	71	MRI	Clinical scores	NA	Microarray	Multivariate Cox proportional hazard models	[136]
	Prediction	104	MRI (T1, T2 CE)	Semantic features	Manual	Microarray (TGCA)	Univariate proportional hazards regression	[137]
	Correlation	18	perfusion CT	Perfusion parameters	Manual	Microarray (TGCA)	Correlation analysis	[138]
	Correlation	46	MRI (DCE, FLAIR)	Semantic features	Manual	Microarray	Kruskal – Wallis H test	[139]
	Prediction	68	MRI (DCE, DWI, anatomy )	Semantic features	Manual	Microarray (TGCA)	Univariate Cox Regression models	[140]
	Correlation	27	MRS	Metabolite concentration	Manual	IHC, PCR	Correlation analysis	[141]
	Correlation	26	MRI (DCE, DWI, DSC, MRS)	Semantic features	Manual	IHC	Correlation analysis	[142]
	Prediction	108	MRI (DCE, DWI)	Semantic features	Manual	Microarray (TGCA)	NA	[143]
LUNG	Prediction	186	CT	Semantic features	Manual	PCR	Univariate analysis and multivariate decision tree models	[144]
	Prediction	138	PET/CT	Shape-based feature, second-order statistics, semantic features	Manual	Microarray	Generalized linear regression with LASSO regularization	[145]
	Correlation Prediction	355	PET/CT	SUV	Manual	Microarray	Student’s t-test, Wilcoxon test, Chi-squared and Fisher’s test	[146]
	Prediction	422	CT	Shape-based features, first-, second- and higher-order statistics	Manual	Microarray	Intraclass correlation coefficient, Friedman test	[36]
KIDNEY	Prediction	70	CT	First-order statistics, semantic features	Manual	Microarray	Multivariate linear regression	[99]
	Correlation	233	CT	Shape-based features, first-order statistics, Semantic features	Manual	DNA-Seq (TCGA)	Fisher’s tests	[147]
	Correlation	103	CT and MRI	Shape-based features, first-order statistics, Semantic features	Manual	Microarray (TCGA)	Pearson’s test and Mann–Whitney *U* test	[148]
	Prediction	58	CT (TCIA)	Shape-based features, first- and second-order statistics	Manual	Microarray (TCGA)	Support vector machine classifier	[149]
LIVER (HCC)	Correlation	30	DCE-CT	Semantic features	NA	Microarray	Correlation analysis	[94]
	Correlation Prediction	47	three-phase contrast enhanced CT	Semantic features	NA	Microarray	Bayesian models	[150]
	Correlation	77	Liver-specific contrast enhanced-MRI	Clinical scores	NA	IHC Microarray	Student’s *t*-test	[151]
PROSTATE	Correlation	45	MRI (T1WI, T2WI, DWI, DCE)	First-order statistics, kinetic parameters, ADC	Manual	IHC	Spearman’s rank correlation coefficient	[152]
	Prediction	17	MRI (T2WI, DWI, DCE)	First-order statistics, kinetic parameters, ADC	Semi-automated	Microarray	Pearson’s correlation, two-way hierarchical clustering	[153]

ADC: apparent diffusion coefficient; DCE: dynamic contrast-enhanced; DSC: dynamic susceptability contrast; DWI: diffusion weithed imaging; IHC: immunohistochemistry; MRS: Magnetic Resonance Spectroscopy; NA: not applicable; T1WI: T1 weighed imaging; T2WI: T2 weighed imaging; T1-c: T1 weighed post contrast.
